# Experiences and Preferences Reported with an At-Home Self-Collection Device Compared with In-Clinic Speculum-Based Cervical Cancer Screening in the United States

**DOI:** 10.1089/whr.2025.0017

**Published:** 2025-05-19

**Authors:** LaShonda Crane, Ashley Jennings, Megan B. Fitzpatrick, Meghna Mukherjee, Callie Pitchford, Amy Nacht, Nia’Ja Mack, Kristina Krueger, Jessica Favreau, Kristin Conway, Laura Gillis, Christine Conageski, Elizabeth Sutton

**Affiliations:** ^1^ Planned Parenthood Gulf Coast, Inc, Houston, Texas, USA.; ^2^ University of Wisconsin, Madison, Wisconsin, USA.; ^3^ Teal Health, Inc, San Francisco, California, USA.; ^4^ Woman’s Hospital, Baton Rouge, Louisiana, USA.; ^5^ University of Colorado School of Medicine, Aurora, Colorado, USA.; ^6^ Crescendo, MD, Portola Valley, California, USA.; ^7^ Beth Israel Deaconess Medical Center, Boston, Massachusetts, USA.; ^8^ NxGen BioStats, LLC, Mount Airy, Maryland, USA.

**Keywords:** HPV test, cervical cancer screening, at-home self-collection, speculum, experiences, preferences, United States

## Abstract

**Background:**

Vaginal self-collection offers an effective option for cervical cancer (CC) screening that can improve screening experiences and engagement.

**Objective:**

This article presents data from a multi-center pilot study (*n* = 185) across the United States, evaluating experiences and preferences reported with an at-home self-collection device compared with clinician-collected screening using a speculum. The device is designed specifically for at-home self-collection and optimized for performance and usability, where collected samples are tested for high-risk human papillomavirus (hrHPV) using the Roche cobas HPV test. In an earlier report, self-collected samples using this device showed high agreement for hrHPV detection when compared with clinician-collection with a speculum and cervical brush.

**Study Design:**

Participants were screened with a self-collection device and a clinician-collection. They provided feedback about their experiences via usability and preference surveys.

**Results:**

Significantly more participants reported pain (*p* < 0.001), discomfort (*p* < 0.001), embarrassment (*p* < 0.001), and nervousness (*p* < 0.001) during clinician-collection compared with self-collection. In contrast, being at-ease (*p* < 0.001) and in-control (*p* < 0.001) were reported significantly more during self-collection. Similar patterns held across demographic groups relevant to CC screening engagement and related risk (i.e., sexual orientation, menopause status, income, and prior HPV diagnosis). Almost all (94% [156/166]) felt confident using the at-home self-collection device if they knew the results would be equivalent to clinician-collection. The device demonstrated wide usability, with 96% (163/170) successfully self-collecting using only the device’s provided instructions.

**Conclusion:**

By improving screening experiences and accessibility, at-home self-collection can increase screening participation and accelerate progress toward eliminating CC as a public health concern in the United States.

## Introduction

Each year in the United States, over 4,000 women die from cervical cancer (CC) with an average of 12,000 new cases.^1^ CC is the third leading cause of cancer death for young women, and incidence has been increasing by 1%–2% annually for those aged 30–44 years.^2–4^ Almost all CCs are caused by high-risk human papillomavirus (hrHPV).^5^ Most of these cancers can be prevented through screening and early detection of precancerous lesions. Prevention can be further improved through more sensitive testing using HPV assays, which enables earlier detection and disease treatment.^6,7^

At least 20 million (or 1 in 4) women and people with a cervix in the United States are behind on their routine CC screenings, and since 2000, screening participation has been steadily declining across racial, ethnic, insurance status, and income groups, leading to premature, preventable deaths.^8–12^ Reasons cited for delayed screening include inadequate insurance coverage, language/cultural barriers, lack of clinic access, competing priorities (*e.g.*, work schedules, other health issues, childcare), financial constraints, lack of HPV, CC, and screening awareness, and negative experiences with clinic-based screening.^10,13^ Robust evidence also shows that sexual trauma and mistrust of clinicians are significant deterrents to clinic-based CC screenings.^14–17^ Disproportionately affected groups in the United States include those without access to care, young women, and those from racial and ethnic minority groups; CC incidence and mortality are increasing in these populations.^18–20^

With routine screening, CC is highly preventable and has a high cure rate of 91% when detected early.^21^ Any increase in screening engagement leads to “clinically meaningful health benefits,” such as avoided cancers, fewer deaths, and life-years gained.^19^ For example, improving CC screening engagement can prevent 93% of annual CC cases in the United States.^22^ Models also highlight that increasing screening engagement combined with HPV vaccination is the most cost-effective and feasible approach to eliminating CC as a public health concern in the United States, accelerating progress toward elimination by 10–13 years.^23,24^

Vaginal self-collected sampling has demonstrated high-relative sensitivity for detecting hrHPV related to cervical dysplasia when compared with clinician-collected samples.^25^ Self-collection also increases accessibility and acceptability of CC screening.^26,27^ In 2014, the U.S. Food and Drug Administration (FDA) approved a primary screening HPV test that has over 92% sensitivity for precancerous cervical dysplasia (vs. 53% for cytology alone).^28^ In 2024, the FDA approved the use of these primary screening HPV tests for self-collection within healthcare settings only. In 2025, self-collection for at-home use was FDA-approved using the device discussed herein.^29,30^ Primary HPV is an optimal screening test for CC, and self-collected HPV samples have similar accuracy to clinician-collection tests while also being associated with increased screening in underscreened populations.^31^ At-home self-collected samples offer benefits from both public health and individual patient standpoints when offered within a comprehensive CC screening program.^32,33^ Many screening barriers can be mitigated by at-home self-collection, and resources can be better allocated to patients at the highest risk of developing CC.

This article presents the acceptability of an at-home vaginal self-collection device compared with the standard clinic-based exam using a speculum. In prior publications from this cohort, the vaginal samples collected with the at-home self-collection device showed high positive (95%) and negative (91%) percent agreement of self-collected HPV samples compared with paired clinician-collected samples.^32^ This article builds on self-collection research, analyzing subgroups relevant to CC screening engagement and related risk (sexual orientation, menopause status, prior HPV diagnosis, and income) and highlighting statistically significant differences in experiences with self-collection compared with clinician-collection.^34^ A user-friendly at-home self-collection could increase screening engagement by improving access and experience.

## Pilot Study Design and Methods

This multi-center method comparison study evaluated the accuracy of an at-home self-collection device and collected usability and preferences feedback.^32^ The study was designed to demonstrate whether individuals who are representative of the intended use population can appropriately use the self-collection device (and accompanying instructions) to collect adequate vaginal samples for use in hrHPV testing. Participants were provided questionnaires related to experiences with self-collection and clinician-collection, which we evaluate here. Participants were recruited between August 2022 and October 2023; once study coordinators informed individuals about the ongoing study, those interested in participating provided written informed consent (ICF) prior to enrollment. This ICF, study protocol, and human subject protections were reviewed and approved by an institutional review board (WCG IRB, TLH-ED-004, ClinicalTrials.gov registration, NCT05669911).

**FIG. 1.**
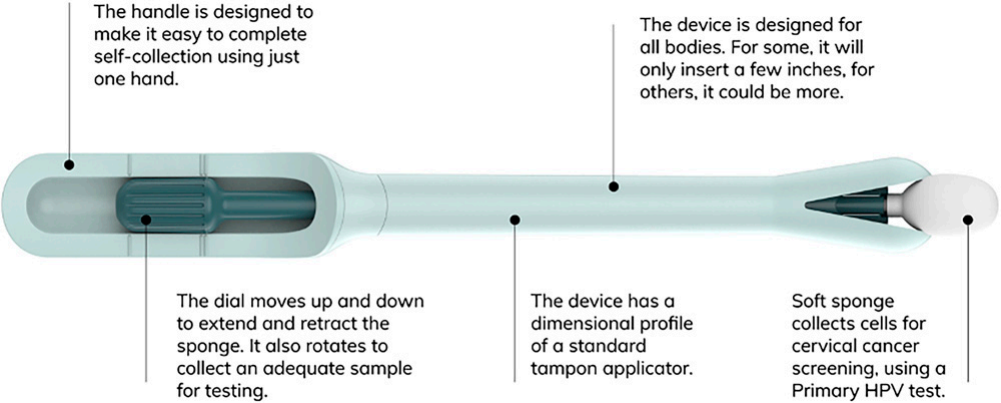
Illustration of the at-home self-collection device studied, with descriptors included for key components and features of the device.

The device is designed for optimal and easy at-home self-collection, and the method is optimized for sample mailing to a laboratory *via* standard postal service (Fig. 1). It is a single-use vaginal applicator, like a tampon, which has a unique tip that expands to expose the sponge collection material. Samples collected with the device are tested using FDA-approved primary HPV assays reflected in medical guidelines for screening (USPSTF, ACS, ACOG).^32^

Respondents were recruited from seven clinical sites in the United States including University of Colorado Hospital (Aurora, CO), University of Wisconsin (Madison, WI), Planned Parenthood Gulf Coast (Houston, TX), Woman’s Hospital (Baton Rouge, LA), and two community sites in Boston, MA, and Portola Valley, CA. Inclusion criteria required ages 25–65 and an intact cervix. Exclusions included pregnancy, menstruation, and application of routinely used vaginal products within 24 hours of study sample collection.

After informed consent and enrollment, respondents were provided the self-collect device and written/pictorial instructions; they then self-collected in a simulated at-home setting. Study coordinators were instructed not to give participants additional instructions or information about using the self-collection device. A clinician-collected research sample followed. Participants also completed two surveys to evaluate usability, preferences, and experiences with both screening methods.

The surveys presented 30 multiple-choice questions about device/instruction usability and 40 multiple-choice options about experiences and preferences around prior clinician-collection screenings and self-collection with the device. Participants were not required to respond to every survey question. Topics included barriers to clinic-based CC screenings and delays in screening due to the following reasons (select none or one or more reasons): financial issues, transportation issues, clinic-related issues, unaware of medical reasons for Pap smear, uncomfortable with the exam, afraid of the results, lack of time, no specific reason/other. Experiences with both collection methods included assessing reports of the following: pain, discomfort, embarrassment, nervousness, neutral, at-ease, and in-control. Narrative comments were also solicited. Usability and preferences are evaluated using descriptive statistics. Analyses from statistical significance tests (*p*-value <0.05) evaluate how experiences vary between self- and clinician-collection. For each positive (at-ease, neutral, in-control) and negative (pain, embarrassment, discomfort, nervousness) experience reported during self- and clinician-collection, McNemar’s exact test is used, which is best suited for paired nominal data. Qualitative data from 53 comments provide further perspective. Comments were coded using an abductive approach, with codes based on quantitative summaries and emergent themes.

## Results

Results focus on experiences with the self-collection device, prior clinician collections, screening preferences, and usability of the self-collection device. A total of 185 individuals were enrolled, and 137 had adequately completed the surveys’ collection experience portion (per McNemar’s analytical method) to be included in the data presented in this article. The parameters of McNemar’s analytical method require matched or paired data. Per McNemar’s method, all subjects who did not have blank or missing responses for all collection experience response options (*i.e.,* selected at least one response in either collection method) are included in the statistical significance analyses (*N =* 137). Where descriptive data are presented (*i.e.,* to evaluate usability and preferences), missing responses are excluded from denominators to reflect valid percentages. Illustrative quotes are included alongside statistical results. Participants represent diverse demographics based on the U.S. screening-aged population (Table 1).

**Table 1.** Participant Demographics

Characteristic	Response category	Number	Percentage
Race	White	113	61.1% (113/185)
	Black or African American	51	27.6% (51/185)
	Asian	9	4.9% (9/185)
	American Indian or Alaska Native	4	2.2% (4/185)
	Other	8	4.3% (8/185)
Ethnicity	Not Hispanic/non-Latinx	161	87.0% (161/185)
	Hispanic/Latinx	21	11.4% (21/185)
Age group	25–29	26	14.1% (26/185)
	30–39	62	33.5% (62/185)
	40–49	50	27.0% (50/185)
	50–65	26	14.1% (26/185)
Menopause status	Premenopausal	133	71.9% (133/185)
	Perimenopausal	29	15.7% (29/185)
	Postmenopausal (menstruation has stopped for at least 12 months)	21	11.4% (21/185)
Location type^a^	Urban (large city)	46	32.6% (46/141)
	Suburban (near large city)	41	29.1% (41/141)
	Small city or town	24	17.0% (24/141)
	Rural (remote from city/town)	7	5.0% (7/141)
Education	Middle school (grade 8) or below	4	2.2% (4/185)
	High school or equivalent (GED)	32	17.3% (32/185)
	Vocational or associate’s degree	35	18.9% (35/185)
	Bachelor’s degree	24	13.0% (24/185)
	Master’s degree or higher	26	14.1% (26/185)
Sexual orientation	Heterosexual/straight	161	87.0% (161/185)
	Gay/lesbian, bisexual, pansexual, other	23	12.4% (23/185)
	Prefer not to reply	1	0.5% (1/185)
HPV vaccination	Yes	70	37.8% (70/185)
	No	102	55.1% (102/185)
Prior HPV diagnosis	Yes	122	65.9% (122/185)
	No	61	33.0% (61/185)
Employment status^a^	Yes, employed (full-time)	76	53.9% (76/141)
	Yes, employed (part-time)	23	16.3% (23/141)
	Not employed (seeking employment)	13	9.2% (13/141)
	Not employed (not seeking employment)	9	6.4% (9/141)
Annual income^a^	Less than $19,999	27	19.1% (27/141)
	$20,000–$49,999	38	27.0% (38/141)
	$50,000–$99,999	29	20.6% (29/141)
	$100,000–$149,999	10	7.1% (10/141)
	More than $150,000	11	7.8% (11/141)

^a^ These demographic characteristics were not recorded for 44 respondents in part 1 of the pilot study.

In this study population, 35% (58/167) report having delayed or avoided CC screening in the past, which is consistent with national statistics.^1^ The three most frequently reported barriers to attending clinic-based screening are lack of time, being uncomfortable with the exam, and financial issues.

Participants report that the self-collection device is usable: 95% (161/169) had no issues opening the device package; 98% (167/169) could hold, position, and guide it into their vagina correctly; and 98% (166/170) reported the instructions were clear. Overall, 96% (163/170) self-collected relying on only the user instructions provided; only one participant reported asking the study coordinator for help. For 96% (161/167), self-collection using the device took under 5 minutes, with 68% (114/167) completing in under 2 minutes. Comments elaborated on usability:

“The instructions were clear, but I read them several times to be confident. The wand was easy to use and the process painless,”

“(Using the device) was easy and quick (and) wasn’t painful.”

“The 1st time […] you have to read instructions quite a few times to make sure you are doing it right. But after doing it is super easy.”

Further, 94% (156/166) are confident using the self-collection device, with the assurance that self-collection would be equivalent to clinician-collected specimens, and 87% (145/166) report a greater likelihood of engaging in routine screening with an at-home option. These findings are consistent across key demographic groups (*e.g.*, race, ethnicity, education, HPV status, insurance status, location).

### Overall experiences with self- and clinician-collected CC screening

The differences in experience reported during self- versus clinician-collection are statistically significant across experience categories, aside from “neutral.” Negative experiences of pain (*p* < 0.001), discomfort (*p* < 0.001), embarrassment (*p* < 0.001), and nervousness (*p* < 0.001) are reported significantly more during clinician-collection (Table 2). Whereas positive experiences of being at-ease (*p* = 0.028) and in-control (*p* < 0.001) are reported significantly more during self-collection.

**Table 2.** Statistical Significance Summary of Overall Self- and Clinician-Collection Feelings (*n* = 137), Including All Participants with Paired Responses for Collection Experiences

Feeling	Percentage responded “yes” (%, *n*/N)		Difference (%)		*p*-value (McNemar’s exact test)
	Self-collect	Clinician-collect	Difference (clinician-self)	95% CI	
Pain	2.2% (3/137)	21.2% (29/137)	19.0%	(11.0%, 26.8%)	*p* < 0.001^*^
Discomfort	19.7% (27/137)	52.6% (72/137)	32.8%	(21.8%, 42.7%)	*p* < 0.001^*^
Embarrassment	2.2% (3/137)	14.6% (20/137)	12.4%	(5.9%, 19.1%)	*p* < 0.001^*^
Nervous	6.6% (9/137)	35.0% (48/137)	28.5%	(18.5%, 37.6%)	*p* < 0.001^*^
At-ease	24.8% (34/137)	13.9% (19/137)	−10.9%	(−20.6%, −1.1%)	0.028^*^
Neutral	25.5% (35/137)	17.5% (24/137)	−8.0%	(−17.6%, 1.6%)	0.102
In-control	36.5% (50/137)	5.8% (8/137)	−30.7%	(−39.7%, −20.7%)	*p* < 0.001^*^

^*^ Indicates statistical significance with *p*-value <0.05.

CI, confidence interval.

Overall, the top three experiences associated with clinician-collection are discomfort, nervousness, and pain. The top three experiences reported with self-collection are feeling in-control, at-ease, and neutral. Notably, there are far fewer reports of pain during self-collection with the device compared with clinician-collection.

This pattern recurs throughout the subgroups analyzed below.

### Sexual orientation and experiences with CC screening

Although those who identify as “Gay/Lesbian, Bisexual, Pansexual, Other” have distinct identities and experiences, we report collectively as “LGBQ+” due to the overall small number of study respondents within each category and given they often share similar experiences around CC screening.

Among the LGBQ+ group, negative experiences of pain, discomfort, and nervousness are reported significantly more during clinician-collection compared with self-collection (Table 3). Positive experiences of neutrality are reported significantly more during self-collection.

**Table 3.** Statistical Significance Summary of Self- and Clinician-Collection Experiences Reported Among LGBQ+ Group (*n* = 22), Including All Participants Who Identified with LGBQ+ and Had Paired Responses for Collection Experiences

Feeling	Percentage responded “yes” (%, *n*/*N*)		Difference (%)		*p*-value (McNemar’s exact test)
	Self-collect	Clinician-collect	Difference (clinician-self)	95% CI	
Pain	4.5% (1/22)	40.9% (9/22)	36.4%	(7.6%, 59.3%)	0.008^*^
Discomfort	27.3% (6/22)	68.2% (15/22)	40.9%	(4.9%, 67.6%)	0.023^*^
Embarrassment	4.5% (1/22)	27.3% (6/22)	22.7%	(−1.0%, 45.4%)	0.063
Nervous	9.1% (2/22)	59.1% (13/22)	50.0%	(14.6%, 73.7%)	0.003^*^
At-ease	22.7% (5/22)	0.0% (0/22)	−22.7%	—	N/A^a^
Neutral	50.0% (11/22)	13.6% (3/22)	−36.4%	(−61.4%, −4.4%)	0.022^*^
In-control	31.8% (7/22)	0.0% (0/22)	−31.8%	—	N/A^a^

^a^ Cannot calculate *p*-value because there is no difference in the proportion of responses between clinician- and self-collection.

^*^ Indicates statistical significance with *p-*value < 0.05.

Overall, the LGBQ+ group reports pain, discomfort, and nervousness most frequently during clinician-collection. To compare, heterosexual individuals report notably lower pain (24%, [27/114]), embarrassment (12%, [14/114]), and nervousness (31%, [35/114]) during clinician-collection.

However, fewer negative experiences are associated with self-collection. In self-collection, 5% (1/22) of LGBQ+ respondents report pain and embarrassment, 27% (6/22) report discomfort, and 9% (2/22) report nervousness. In contrast, 32% (7/22) report feeling in-control and 23% (5/22) at-ease during self-collection (none report at-ease or in-control experiences during clinician-collection). Finally, 50% (11/22) report neutral experiences during self-collection, compared with 14% (3/22) who report neutrality during clinician-collection.

### Menopause status and experiences with CC screening

Postmenopausal refers to women whose menstrual periods have stopped permanently (for at least 12 months), while perimenopausal refers to those who are gradually experiencing menopause-related symptoms. These McNemar’s test values reflect 21 perimenopausal and 13 postmenopausal respondents.

In clinician-collection, pain, discomfort, and nervousness are reported most frequently in both menopause status groups. In terms of pain, 46% (6/13) of the postmenopausal group and 24% (5/21) of perimenopausal participants report pain during clinician-collection. Discomfort is reported similarly by those who are perimenopausal (57% [12/21]) and postmenopausal (62% [8/13]). Positive experiences (*i.e.*, in-control, at-ease) associated with clinician-collection are reported less frequently across all menopause status groups.

In contrast, positive experiences are reported more frequently with self-collection than during clinician-collection. There were 39% (5/13) of postmenopausal and 57% (12/21) of perimenopausal individuals who felt in-control during self-collection. Negative experiences are still reported, but less frequently compared with clinician-collection. Reports of pain and discomfort are notably lower during self-collection. No perimenopausal respondents reported pain (vs. 24% [5/21] clinician-collection) and only 8% (1/13) of postmenopausal respondents (vs. 46% [6/13]) reported pain during self-collection. Similarly, 10% (2/21) of perimenopausal (vs. 57% [12/21] clinician-collection) and 46.0% (6/13) of postmenopausal individuals (vs. 62% [8/13]) experienced discomfort during self-collection.

The following comments provide additional perspective:

“(There was) pressure on my cervix but not as much discomfort and no pain or pinching.” (perimenopausal respondent)

“(Self-collection) was very easy and painless.” (perimenopausal respondent)

The lower reports of discomfort during self-collection are statistically significant in the perimenopausal group (*p* = 0.006). Further, significantly more perimenopausal respondents (*p* = 0.001) experienced being in-control during self-collection compared with clinician-collection.

### Prior HPV diagnosis and experiences with CC screening

We analyzed the experiences of participants who had a prior HPV diagnosis. All negative experiences are reported significantly more frequently during clinician-collection compared with self-collection (Table 4). For positive experiences, there is a significant difference in being “in-control” reported more during self-collection.

**Table 4.** Statistical Significance Summary of Self- and Clinician-Collection Experiences Reported Among Participants with Prior HPV Diagnosis (*n* = 107), Including All Participants Who Had Prior HPV Diagnoses and Paired Responses for Collection Experiences

Feeling	Percentage responded “yes” (%, *n*/*N*)		Difference (%)		*p*-value (McNemar’s exact test)
	Self-collect	Clinician-collect	Difference (clinician-self)	95% CI	
Pain	2.8% (3/107)	24.3% (26/107)	21.50%	(11.8%, 30.8%)	*p* < 0.001^*^
Discomfort	19.6% (21/107)	53.3% (57/107)	33.60%	(21.3%, 44.5%)	*p* < 0.001^*^
Embarrassment	2.8% (3/107)	17.8% (19/107)	15.00%	(6.9%, 23.1%)	*p* < 0.001^*^
Nervous	8.4% (9/107)	40.2% (43/107)	31.80%	(19.7%, 42.6%)	*p* < 0.001^*^
At-ease	25.2% (27/107)	15.0% (16/107)	−10.30%	(−21.2%, 0.8%)	0.071
Neutral	26.2% (28/107)	21.5% (23/107)	−4.70%	(−16.1%, 6.8%)	0.487
In-control	41.1% (44/107)	7.5% (8/107)	−33.60%	(−44.3%, −21.7%)	*p* < 0.001^*^

^*^ Indicates statistical significance with *p-*value < 0.05.

Overall, we see that those who have had a previous HPV diagnosis report more negative experiences associated with clinician-collection. The top three experiences associated with clinician-collection are discomfort, nervousness, and pain.

Negative experience reports are lower during self-collection. The top three experiences associated with self-collection are being in-control, at-ease, and neutral. Self-collection with the device brings more positive experiences for those with a prior positive HPV diagnosis.

### Annual income and experiences with CC screening

Across all self-reported income groups, negative experiences around pain, discomfort, and embarrassment were featured more frequently with clinician-collection experiences. Discomfort is reported by 55% or more individuals across income groups. Those in the $20,000–$49,999 income group report discomfort associated with clinician-collection most often (63% [24/38]). Positive experiences of being in-control and at-ease are less frequently associated with the clinician-collection experience across income groups. None of those earning less than $19,999, and fewer than 3% (1/38) earning $20,000–$49,999, associate being in-control with clinician-collection. In contrast, self-collection brings more positive experiences, where all groups report being in control to some extent. Negative experiences with self-collection are less common compared with clinician-collection.

Among lower- and middle-income groups, we find that these differences in experiences reported with self- and clinician-collection are statistically significant in some instances (Table 5). There is a statistically significant difference in discomfort and nervousness being reported more frequently during clinician-collection compared with self-collection in the lower-income group (<$19,999), while all negative experiences are reported significantly more during clinician-collection in the $20,000–$49,999 group. In the middle-income group ($50,000–$99,999), discomfort is reported significantly more frequently during clinician-collection. Being in-control is reported significantly more during self-collection within the $20,000–$49,999 and $50,000–$99,999 groups.

**Table 5.** Statistical Significance Summary of Self- and Clinician-Collection Experiences Across Income Groups (*n* = 115), Including All Participants Who Had Reported Income and Paired Responses for Collection Experiences

Income group	Feeling	Percentage responded “yes” (%, *n*/*N*)		Difference (%)		*p*-value McNemar’s exact test
		Self-collect	Clinician-collect	Difference (clinician-self)	95% CI	
Less than $19,999 (*n* = 27)	Pain	7.4% (2/27)	29.6% (8/27)	22.2%	(−1.6%, 44.2%)	0.070
	Discomfort	29.6% (8/27)	55.6% (15/27)	25.9%	(1.0%, 48.1%)	0.039^*^
	Embarrassment	0.0% (0/27)	11.1% (3/27)	11.1%	—	N/A^a^
	Nervous	3.7% (1/27)	40.7% (11/27)	37.0%	(11.3%, 57.6%)	0.002^*^
	At-ease	37.0% (10/27)	11.1% (3/27)	−25.9%	(−51.5%, 3.7%)	0.092
	Neutral	22.2% (6/27)	18.5% (5/27)	−3.7%	(−30.8%, 23.9%)	1.000
	In-control	37.0% (10/27)	0.0% (0/27)	−37.0%	—	N/A^a^
$20,000–$49,999 (*n* = 38)	Pain	2.6% (1/38)	18.4% (7/38)	15.8%	(1.0%, 31.3%)	0.031^*^
	Discomfort	18.4% (7/38)	63.2% (24/38)	44.7%	(18.4%, 64.7%)	*p* < 0.001^*^
	Embarrassment	2.6% (1/38)	23.7% (9/38)	21.1%	(4.3%, 37.3%)	0.008^*^
	Nervous	5.3% (2/38)	42.1% (16/38)	36.8%	(15.9%, 54.0%)	*p* < 0.001^*^
	At-ease	18.4% (7/38)	13.2% (5/38)	−5.3%	(−25.6%, 15.4%)	0.774
	Neutral	21.1% (8/38)	7.9% (3/38)	−13.2%	(−29.6%, 3.1%)	0.125
	In-control	47.4% (18/38)	2.6% (1/38)	−44.7%	(−62.7%, −21.0%)	*p* < 0.001^*^
$50,000–$99,999 (*n* = 29)	Pain	0.0% (0/29)	24.1% (7/29)	24.1%	—	N/A^a^
	Discomfort	24.1% (7/29)	58.6% (17/29)	34.5%	(4.4%, 58.7%)	0.021^*^
	Embarrassment	3.4% (1/29)	17.2% (5/29)	13.8%	(−3.1%, 31.7%)	0.125
	Nervous	17.2% (5/29)	44.8% (13/29)	27.6%	(−0.8%, 51.9%)	0.057
	At-ease	27.6% (8/29)	24.1% (7/29)	−3.4%	(−27.1%, 20.5%)	1.000
	Neutral	37.9% (11/29)	34.5% (10/29)	−3.4%	(−28.8%, 22.4%)	1.000
	In-control	34.5% (10/29)	10.3% (3/29)	−24.1%	(−45.2%, −1.0%)	0.039^*^
$100,000–$149,999 (*n* = 10)	Pain	0.0% (0/10)	10.0% (1/10)	10.0%	—	N/A^a^
	Discomfort	20.0% (1/10)	60.0% (6/10)	40.0%	(−8.8%, 73.8%)	0.125
	Embarrassment	10.0% (1/10)	20.0% (2/10)	10.0%	(−25.9%, 44.5%)	1.000
	Nervous	0.0% (0/10)	40.0% (4/10)	40.0%	—	N/A^a^
	At-ease	30.0% (3/10)	10.0% (1/10)	−20.0%	(−55.6%, 20.6%)	0.500
	Neutral	30.0% (3/10)	30.0% (3/10)	0.0%	(−46.8%, 46.8%)	1.000
	In-control	60.0% (6/10)	10.0% (1/10)	−50.0%	(−81.3%, 2.2%)	0.063
More than $150,000 (*N* = 11)	Pain	0.0% (0/11)	27.3% (3/11)	27.3%	—	N/A^a^
	Discomfort	27.3% (3/11)	54.5% (6/11)	27.3%	(−13.6%, 61.0%)	0.250
	Embarrassment	0.0% (0/11)	9.1% (1/11)	9.1%	—	N/A^a^
	Nervous	0.0% (0/11)	27.3% (3/11)	27.3%	—	N/A^a^
	At-ease	27.3% (3/11)	18.2% (2/11)	−9.1%	(−41.3%, 23.8%)	1.000
	Neutral	36.4% (4/11)	27.3% (3/11)	−9.1%	(−47.7%, 32.2%)	1.000
	In-control	36.4% (4/11)	27.3% (3/11)	−9.1%	(−47.7%, 32.2%)	1.000

^a^ Cannot calculate *p*-value because there is no difference in the proportion of responses between clinician- and self-collection.

^*^ Indicates statistical significance with *p*-value <0.05.

## Discussion

Participants in this pilot study comparing at-home vaginal self-collection to clinician-collection report more positive experiences about self-collection and find the collection process and device easy to use. Turning to usability and preferences, 96% (163/170) self-collected using only the provided instructions, and for 96% (161/167), the self-collection took under 5 minutes. 94% (156/166) report confidence in using the self-collection device if they knew it would be equivalent to clinician-collected specimens, and 87% (145/166) report they would be more likely to engage in routine screening given an at-home option.

Positive experiences are more frequently associated with self-collection, while negative experiences are observed more during clinician-collection; these associations are statistically significant in nearly all of the analyses conducted. Overall, being at-ease (*p* = 0.028) and in-control (*p* < 0.001) are reported significantly more during self-collection (Table 2). In contrast, pain (*p* < 0.001), discomfort (*p* < 0.001), embarrassment (*p* < 0.001), and nervousness (*p* < 0.001) are reported significantly more during clinician-collection. The greater association of negative experiences with clinician-collection, the existing standard of care, can be a deterrent to routine screening participation and thereby can increase the incidence of late-stage CC diagnoses.^10,13^

Self-collection offers individuals more choices and control over their screening experience. Furthermore, self-collection in the at-home environment can overcome the most persistent barriers to screening including time and financial constraints, lack of clinic access, negative experiences, and trauma.^3,35–37^ Research supports a high rate of engagement with follow-up care (*e.g*., cytology triage, colposcopy) when self-collection is used—85%–94% among those who tested positive for HPV—suggesting that improved screening experiences may also contribute to positive downstream impacts in adherence to follow-up care.^38^ New screening methods should focus on improving the screening experience to increase screening engagement.^15,37,39^

Self-collection is a clinically validated and highly acceptable method to increase screening participation by overcoming some common barriers cited among those who do not participate.^26^ In a meta-analysis of seven studies, comprising 1,470 women, 97% of these respondents found self-collection acceptable.^37^ Acceptability is also high in low-resourced settings and among vulnerable populations such as female-to-male trans patients.^40,41^

Individuals prefer being in control and having choices around their CC screening options, which heightens screening engagement.^15,37^ Our data support these findings, with over six times more reports of being in-control during self-collection than clinician-collection and the difference being statistically significant (*p* < 0.001). Further, pain is a common negative experience with clinician-collected CC screenings.^39^ In our study, pain was reported almost 10 times more frequently compared with self-collection and the difference is statistically significant (*p* < 0.001).

We examined experiences stratified by sexual orientation because LGBQ+ groups experience higher rates of sexual trauma and more systemic implicit bias from health care providers, both of which are barriers to standard clinic-based speculum exams and result in health care disparities.^42,43^ For the LGBQ+ group, all negative experiences (except for embarrassment) are reported significantly more with clinician-collection compared with self-collection. Positive experiences are reported less or not reported at all in association with their clinician-collection experience. CC screening experiences are significantly more positive for self-collection among those who identified as LGBQ+ in our study, highlighting that self-collection has the potential to improve health equity for these individuals who are more often under- or never-screened (and therefore at higher risk of CC).^44^

Analyzing menopause status enables insight into how screening experiences may vary throughout a person’s life stages and physiological changes. Among peri- and postmenopausal respondents in this study, negative experiences are more commonly associated with clinician-collection but lower for self-collection. Pain is a common reason behind postmenopausal individuals (typically older females) avoiding speculum-based CC screenings, as it is often related to physiological changes associated with age and menopause.^10,39^ Our data show that pain reports are almost absent with self-collection (perimenopausal: 0% vs. 24% [5/21]; postmenopausal: 8% [1/13] vs. 46% [6/13]) and reports of discomfort are significantly lower for the perimenopausal group (*p* = 0.006) during self-collection. Improving this experience can increase screening engagement when physiological changes take place.^45^ Regarding preferences, among postmenopausal individuals, 95% (20/21) report they would be more likely to get routine CC screening with at-home self-collection, and 100% (21/21) would be confident doing so if they could be reassured of result equivalency.

Individuals with a prior HPV diagnosis have a higher risk of developing CC and typically need more frequent screenings. Increased screening frequency exposes one to more screening experiences that may deter individuals from the clinic-based exam. Their screening experience may also be shaped by the stigma around sexually transmitted infections, HPV’s association with cancer, as well as associated discrimination.^46^ People with prior HPV diagnoses in this study report all negative experiences significantly more during clinician-collection compared with self-collection. Accordingly, enabling screening engagement through approaches that engender positive experiences (*i.e.*, self-collection) is especially important among this higher-risk group.

Finally, we analyze experiences by income groups because financial barriers are often reported as a reason for delayed CC screenings. While health insurance status is an important factor in how individuals access preventive screenings, financial barriers span beyond insurance status. When it comes to in-clinic CC screening, challenges around obtaining time off work, affording childcare, or securing transportation to attend an in-person appointment for screening can be pertinent for those of lower income with less access to these resources.^35,47^ In our data, the experiences of those at lower-income levels are substantially more positive with self-collection. Further, negative experiences are less common during self-collection. Of note, our analysis finds statistically significant differences in negative experiences being reported more frequently during clinician-collection compared with self-collection among lower-income groups.

Improved experiences, including at-home self-collection, can increase screening engagement, particularly in lower and middle-income groups: 82%–84% of those in income groups below $19,999 (22/27) to $49,999 (32/38) indicate a greater likelihood to screen with an at-home self-collect option, with that value increasing to 93% (27/29) in the $50,000–99,999 group. Due to barriers around resource access, as discussed above, these groups can often be among the never- or underscreened population, putting them at higher risk of CC.^35,47^ As such, an improved screening experience, leading to higher engagement, could be a boon to public health outcomes.

These data show that self-collection improves CC screening experiences and emphasize the importance of offering at-home self-collection options to improve screening engagement, particularly among underscreened or higher-risk subgroups whose experiences have not previously received as much attention (*i.e.*, menopause status, sexual orientation, prior HPV diagnosis). Self-collection is associated with more positive experiences across various groups at higher risk of being underscreened, highlighting the importance of offering self-collection to close the screening gap. Strengths include that participants reflect a diverse demographic sample, representative of the U.S. screening-aged population, and that the sample is robust for studies on experiences (*N =* 137, Table 2), usability, and preferences (*N =* 185, Table 1). Limitations include that not all participants provided responses to all questions, thereby reducing available data in some analyses, particularly when using McNemar’s method. Secondly, some subgroups had a smaller sample size, and although appropriate statistical analyses were conducted, further analyses with larger samples may provide additional insights. Building on this pilot study, a larger pivotal study comparing the at-home self-collection device to clinician-collection has been completed with reports pending (NCT06120205).

## Conclusion

Participants who used a self-collection device designed for at-home use to collect a vaginal sample in this study reported significantly more positive screening experiences and preferred this user-friendly option over clinician-collection. Negative experiences were reported significantly more during clinician-collection, which can deter individuals from routine screening engagement. The majority of participants reported an increased likelihood of screening participation with a user-friendly self-collection option, which demonstrates that improving screening experiences and accessibility can translate to increased engagement in preventive care, particularly among underscreened and/or higher-risk subgroups included in this study (*e.g.*, LGBQ+, prior HPV diagnosis, low-income, peri- and postmenopausal). Given the comparable clinical accuracy to clinician-collection, self-collected HPV tests, particularly those that are user-friendly and designed for at-home use, are a crucial CC screening option that can further optimize screening engagement and public health outcomes.^31,32^

## Abbreviations Used

ACOG: American College of Obstetricians and Gynecologists
ACS: American Cancer Society
CA: California
CC: cervical cancer
CI: confidence interval
CO: Colorado
FDA: Food and Drug Administration
HPV: Human Papillomavirus
HrHPV: High-risk Human Papillomavirus
ICF: informed consent form
IRB: Institutional Review Board
LA: Louisiana
LGBQ+: Lesbian, Gay, Bisexual, Queer+
MA: Massachusetts
TX: Texas
U.S.: United States
USPSTF: United States Preventive Services Task Force
WI: Wisconsin
